# Evaluation of the Antibacterial Activity of *Pleurotus spp.* Cultivated on Different Agricultural Wastes in Chiro, Ethiopia

**DOI:** 10.1155/2020/9312489

**Published:** 2020-08-27

**Authors:** Getachew Gashaw, Amare Fassil, Fuad Redi

**Affiliations:** ^1^College of Natural and Computational Sciences Department of Biology, Oda Bultum University, P. O. Box: 226, Asebe Teferi, Ethiopia; ^2^College of Natural and Computational Sciences Department of Statistics, Oda Bultum University, P. O. Box: 226, Asebe Teferi, Ethiopia

## Abstract

In the present study, mushrooms, *Pleurotus ostreatus* and *Pleurotus florida*, were cultivated on different agricultural wastes namely coffee straw (CS), pea straw (PS), *Sorghum* Grain Residue (SGR), and Wheat Grain (WG) for the evaluation of antibacterial activity. Antimicrobial activity evaluation was carried out against human pathogenic microorganisms, namely, *Escherichia coli*, *Bacillus subtilis*, *Streptococcus faecalis*, *Pseudomonas aeruginosa*, and *Salmonella typhi* by using the disc diffusion method. Methanolic extracts of *P. ostreatus* cultivated on a S*orghum* grain residue substrate were recorded for the highest antibacterial activity against *E. coli* (19.8 mm) and *P. aeruginosa* (16.4 mm), and methanolic extracts of *P. florida* cultivated on a wheat grain substrate were recorded for the highest antibacterial activity against *E. coli* (18.6 mm) and *S. faecalis* (14.8 mm). Therefore, results suggested that *P. ostreatus* and *P. florida* cultivated on the coffee straw and *Sorghum* grain substrate were found with the highest antimicrobial activity in comparison to other substrates. The results supported that the methanolic extracts of *P. ostreatus* and *P. florida* might indeed be potential sources of antibacterial agents.

## 1. Introduction

### 1.1. Background and Justification

Mushrooms are a nutritious food source, being rich in protein, vitamins, and minerals. They are also known to contain substances that enhance the immune system, fight infectious diseases, and lower blood pressure and cholesterol levels. Mushroom is being used as a valuable food source and traditional medicine around the world since ancient times, especially in China and Japan. Mushrooms are rich sources of bioactive compounds such as ß-glucan, proteoglucan, lectin, phenolic compounds, flavonoids, polysaccharides, triterpenoids, diatery fibre, lentinan, schizophyllan, lovastatin, pleuran, steroids, glycopeptides, terpenes, saponins, xanthones, coumarins, alkaloid, purin, purimidin, kinon, fenil propanoid, kalvasin, volvotoksin, flammutoksin, porisin, AHCC, maitake D-fraction, ribonucleas, and eryngeolysin. Pharmacological and nutritional aspects make mushroom as an important tool for the ailment of severe diseases such as microbial and viral infections, cancer, tumor, inflammation, cardiovascular diseases, and immunomodulating diseases [1].

*Pleurotus* spp. is promising as medicinal mushroom, exhibiting hematological, antiviral, antitumor, antibiotic, antibacterial, hypocholesterolic, and immunomodulation activities [2]. The oyster mushroom may also be considered as medicinal mushroom for its hypocholesterolic property because it contains statins such as lovastatin which reduces cholesterol [3].

The oyster mushroom, *Pleurotus* spp., is widely cultivated on a wide range of substrates which are composed of lignin and cellulose. Cultivation of *Pleurotus* spp. supports a broad range of temperatures (15–30°C) on different ranges of substrates such as agro waste residues, weeds, and wastes after the production of food, feed, vitamins, enzymes, and a number of pharmaceuticals in addition to their waste degradation and detoxification properties [4, 5]. The bioactive compounds present in *Pleurotus* spp. make it a medicinally important mushroom [4].

In the recent years, high scale usage of synthetic antibiotic has led to the emergence of multidrug resistance pathogens and is now posing a threat to the world. Therefore, a search for natural plant-based antimicrobial agents is in need. This development is the consequence of the limited effectiveness of synthetic products to fight against newer and drug-resistant bacteria. For this purpose, the antimicrobial properties of many natural compounds from a wide variety of plant species have been assessed [6]. Therefore, this study was conducted to evaluate the effect of different substrates on the antimicrobial activity of *P. ostreatus* and *P. florida*.

In Ethiopia, no studies have been conducted for the evaluation of the antibacterial activity of *Pleurotus* spp. cultivated on different agricultural wastes, and little is known of the biology and potential antibacterial sources of *Pleurotus* species grown on different agricultural waste in spite of its nutritional importance. Hence, due to this importance, attention needs to be given to the antibacterial agents which could serve as an input for efficiently managing bacterial disease. Therefore, this study was undertaken with the objective of evaluation of the antibacterial activity of *Pleurotus* species grown on different agricultural wastes in the West Hararge Zone, in Chiro, Ethiopia.

## 2. Materials and Methods

### 2.1. Chemicals and Reagents

Methanol, ethanol and aqueous solutions, streptomycin, nutrient agar, Cezpek Dox agar, Mueller Hinton agar, and Mueller Hinton broth.

### 2.2. Agricultural Wastes Utilized

Coffee straw, *Sorghum* grain residue, pea straw, and wheat grain.

### 2.3. Spawn Collection

The mother spawn of *Pleurotus florida* and *Pleurotus ostreatus* were collected which were cultivated and sold as edible mushrooms from Addis Ababa University, Ethiopia.

### 2.4. Spawn Preparation

Spawn was prepared by using method of Bano and Shrivastava [7] with slight modifications. One kg of wheat grain was cooked for 40 min and, after that, washed in tap-water. Grain was drained and supplemented with 2 g lime and 8 g gypsum and mixed manually. Then, grain was filled in poly propylene (PP) bags of 1 kg capacity and sterilized in an autoclave at 121ºC for 15 min. After cooling, a PP bag was inoculated with freshly prepared mycelium (previously prepared PDA plate) and incubated at 25ºC for two weeks in an incubator.

### 2.5. Preparation of the Mushroom Extract

The present study was carried out to know the antimicrobial activity of *Pleurotus* spp. (*P*. *ostreatus* and *P.florida*) mushrooms cultivated on different agricultural wastes. Freshly harvested fruiting bodies from *P. ostreatus* and *P. florida* were shade-dried and finely powdered. Twenty grams of the powder was extracted with 200 ml of 95% solvent methanol, ethanol, and aqueous separately using a soxhlet apparatus at 25°C at 150 rpm for 24 hours and filtered through Whatman no. 4 paper. The remaining extract was filtered and evaporated by vacuum distillation; the filtrate, thus, obtained was used as mushroom extract [8]. For the entire analysis, compounds of the extract were dissolved in dimethylsulfoxide (DMSO), and filter-sterilization was carried out through a 0.22-*μ*m membrane filter. Extracts were kept in the dark at 4°C for not more than 1 week prior to use. The extraction was repeated twice.

### 2.6. Pathogenic Microorganisms Used for the Antimicrobial Assays

In this experiment, five bacterial strains were used for antimicrobial assay. The preserved strains were obtained from the Addis Ababa University Microbiology Department. The pathogens were maintained on nutrient agar, Cezpek Dox agar, Mueller Hinton agar, and Mueller Hinton broth.

#### 2.6.1. Antibacterial Assay

The antibacterial activity of the methanolic extract of mushrooms was determined by the agar well diffusion method [9, 10] with slight modification to suit the conditions of this experiment. Briefly, the methanol extracts were dissolved in 3% dimethylsulfoxide (DMSO) to a final concentration of 10 mg/ml and filter sterilized through a 0.45-*μ*m membrane filter. Small wells (6 mm in diameter) were made in the agar plates by using a sterile cork borer. 20–100 microliters of the extract of each isolate of mushrooms was loaded into the different wells. An overnight culture of each microbial isolate was emulsified with nutrient broth to a turbidity that was equivalent to 0.5 McFarland (10^8^ cfu/ml). In order to determine the antimicrobial efficacy of the fractions, an aliquot of the test culture (100 *μ*l) was evenly spread over the surface of the solidified agar. Bacteria were cultured on Mueller Hinton agar. Streptomycin (20–100 *μ*l/well) was used as positive control for test microorganisms. All the preloaded plates with the respective extract and test organism were incubated at 37°C, for 24 hours. After the incubation period, the zones of inhibitions were measured in millimeters [11].

### 2.7. Minimum Inhibitory Concentration (MIC)

The standard agar dilution protocol with doubling dilution was used. The extract was incorporated into nutrient agar at concentrations ranging from 200 *µ*l to 1000 *µ*l. A control without the extract was also prepared. 10 *μ*L of each test organisms, previously diluted to 10 CFU/ml, was used to inoculate the plates. These were incubated at 37°C for 24 h in the first instance and for another 24 h before the growth was observed and recorded. The minimum inhibitory concentrations (MICs) of the extract for each test microorganism were considered the agar plate with the lowest concentrations without growth [12].

### 2.8. Data Entry and Analysis

All data were analyzed and expressed as mean ± standard deviations of three separate determinations (*n* = 3). The statistical analysis was carried out by using SAS for Windows, version 20.0. One-way analysis of variance (ANOVA) and LSD comparisons were carried out to detect the significant difference (*p* < 0.05) between the mean values that had more than two groups.

## 3. Result and Discussion

### 3.1. Antibacterial Effects of Various Extracts against Pathogenic Microorganisms

The antimicrobial activity of *Pleurotus* spp. (*P. ostreatus* and *P. florida*) was carried out against pathogenic microorganisms, namely, *Escherichia coli*, *Bacillus subtilis, Streptococcus faecalis*, *Pseudomonas aeruginosa,* and *Salmonella typhi*.

### 3.2. Effects on *E. coli*

The methanolic, ethanolic, and aqueous extracts of mushroom species have shown antibacterial effects on the growth of the cultures of *E. coli*. Furthermore, the antibiotic effect of methanolic and ethanolic extracts of *P. ostreatus* and *P. florida* is more than that of the aqueous extracts of the two species. In addition, it was also found that their effects with all extracts were found more on the methanolic extract than the others. A higher effect on the methanolic extract than on the others may be due to the higher diffusion rate of the extract into the methanolic than in the others as the solid disc of +ve control has variation with these effects. The antibiotic effect of all extracts is not because of individual secondary metabolites but is the combined effect of all metabolites, as mentioned in Tables 1–3.

The maximum activity recorded against *E. coli*. was 19.8 mm showed by the methanolic extract of *P. ostreatus* when cultivated on the *Sorghum* grain residue substrate and the minimum was 7.2 mm by the aqueous extract, as shown in Tables 1 and 3. The obtained results showed similarity with the findings of Menaga et al. [13]. In the present study, methanolic extracts of *Pleurotus* spp. showed the activity against *E. coli*. (7.3 mm–19.8 mm), *B. subtilis* (7.1 mm–15.5 mm), *Streptococcus faecalis* (6.9 mm–14.8 mm), *Pseudomonas aeruginosa* (8.7 mm–16.4 mm), and *Salmonella typhi* (4.7 mm–16.9 mm), as shown in Tables 1, 4–7. On the other hand, ethanolic extracts showed the antimicrobial activity against *E. coli*. (8.3 mm–18.4 mm), *B. subtilis* (7.3 mm–15.3 mm), *Streptococcus faecalis* (6.6 mm–13.9 mm), *Pseudomonas aeruginosa* (8.4 mm–15.6 mm), and *Salmonella typhi* (3.2 mm–15.4 mm), as given in Tables 2, 8–11. Mushrooms obtained from the coffee straw and *Sorghum* grain residue substrate were found with an excellent antimicrobial activity, whereas the mushrooms obtained from the pea straw and wheat grain substrate were recorded with a poor antimicrobial activity because of low production of bioactive compounds. Methanolic extracts of *P. ostreatus* from the *Sorghum* grain residue substrate gave best results against *E. coli* (19.8 mm), and methanolic extracts of *P. florida* from *Sorghum* grain gave best results against *B. subtilis* (15.5 mm) and *streptococcus faecalis* (14.8 mm).

On the other hand, aqueous extracts showed the antimicrobial activity against *E. coli* (7.2 mm–17.4 mm), *B. subtilis* (6.3 mm–14.7 mm), *Streptococcus faecalis* (6.2 mm–14.5 mm), *Pseudomonas aeruginosa* (7.1 mm–15.2 mm), and *Salmonella typhi* (4.6 mm–14.5 mm), as given in Tables 3, 12–15. Mushrooms obtained from the coffee straw and *Sorghum* grain substrate were found with an excellent antimicrobial activity, whereas the mushrooms obtained from the pea straw substrate were recorded with a poor antimicrobial activity because of low production of bioactive compounds.

The results of Akyuz and Kirbag [18] on the antimicrobial activity of the methanolic extract of *Pleurotus* spp. against *B. megaterium, E. coli, K. pneumonia, S. aureus, C. albicans, C. glabrata Epidermophyton* spp., and *Trichophyton* spp. explained that petroleum ether and acetone extracts of *P. ostreatus* were found effective against *Staphylococcus spp*. (7.0–7.6 mm), *Bacillus spp*. (7.1–7.8 mm), *S. thyphi* (7.0–7.5 mm), *E. coli* (7.0–8.2 mm), *K. pneumoniae* (7.0–7.1 mm), and *Candida spp.* (8.0–8.3 mm). *P. ostreatus* showed a high activity against *C.glabrata* of 15.5 mm. Jagadish et al. [19] reported that the ethanol extract of *P. florida* and *P. aureovillosus* did not exhibit an antimicrobial effect against *K. pneumoniae*, *P. vulgaris, P. aeruginosa*, and *C. albicans*, but showed activity against *S. aureus* (16.0 mm and 20.0 mm), *S. mutans* (14.0 and 17.0 mm), *M. luteus* (16.0 and 19.0 mm), *B. subtilis* (9.0 and 14.0 mm), and *E. coli* (12.0 and 14.0 mm), respectively. Iwalokun et al. [1] also reported the similar results. Mondal et al. [16] found that the inhibition zone ranging from 3.5 mm–20 mm was formed by the extract of *P .ostreatus* during an antimicrobial study. Its methanolic extracts gave the best results against *E. coli* (15.2 mm), and *S. aureus* (16.6 mm) was very close to the present study. Surekha et al. [17] reported the antimicrobial activity of *P. ostreatus* against pathogenic bacteria *E. coli* (15 mm), *S. aureus* (24 mm), and *P. vulgaris* (18 mm). Thillaimaharani et al. [18] reported that the antibacterial activity of different extracts of *P. Florida* was tested against 8 human bacterial pathogens, namely, *E. coli*, *S. typhi*, *K. pneumoniae*, *V. parahaemolyticus*, *K. oxytoca*, *P. murabilus*, *V. cholarae*, and *Streptococcus* spp., and the antibacterial activity of the ethanol extract of *P. florida* was found maximum (23 mm) against *Streptococcus spp*. and minimum (4 mm) against *V. parahaemolyticus.* The antimicrobial activity against *E. coli* was found to be 11 mm. Akyuz and Kirbag [18] reported the same results for the ethanol extract of *P. eryngii* that it showed the maximum antifungal activity against *C. albicans* (7.7 mm), *C. glabrata* (7.7–9.3 mm), *Epidermopyton* sp. (7.7–8 mm), and *Trichophyton* spp. (7.7–8.7 mm).

A previous study of Menaga et al. [13] on the antimicrobial activity of the ethanolic extract of *P. florida* exhibited the highest activity against *Pseudomonas* spp. and *Campylobacter* spp., whereas the methanol extract showed higher activity against the *E. coli, Salmonella typhi, Staphylococcus aureus, Camphylobacter* sp., and *Vibrio* sp. aqueous extract, also revealing a high zone formation against *Vibrio* sp. of 24 mm. Menaga et al. [13] concluded that the methanol extract showed an activity against *E. coli* (21 mm)*, Salmonella typhi* (20 mm)*, Staphylococcus aureus* (20 mm)*, Camphylobacter* spp. (19 mm)*, Bacillus* spp. (14 mm)*, Pseudomonas* spp. (8 mm)*, Klebsiella* spp. (12 mm), and *Vibrio* spp. (20 mm). In a previous study, Jonathan [5] reported that the sporophore methanolic extract of *Pleurotus florida* showed an activity in *E. coli* (13 mm) and *Klebsiella* spp. (20 mm) and no activity against *Bacillus* spp., *Pseudomonas*, and *Proteus* spp. (30 mm). Menaga et al. [13] reported the zone formation in *Pseudomonas* spp., (20 mm), *Salmonella* spp., (20 mm), and *Klebsiella pneumonae* (13 mm), whereas the ethanolic extract of mycelium showed zone formation in *Staphylococcus aureus* (16 mm), *Streptococcus mutans* (14 mm), *Escherichia coli* (12 mm), *Micrococcus luteus* (16 mm), and *Bacillus subtilis* (9 mm) and no zone formation against *Pseudomonas aeruginosa*, *Salmonella abony*, *Klebsiella pneumoniae*, *Proteus vulgaris*, and *Candida albicans*.

### 3.3. Effects on *Bacillus subtilis*

The methanolic, ethanolic, and aqueous extracts of mushroom species have shown antibacterial effects on the growth of the cultures of *B. subtilis.* The maximum activity was recorded against *B. subtilis* with 15.5 mm showed by the methanolic extract of *P. florida* when cultivated on the SGR substrate, and the minimum was 6.3 mm by the aqueous extract, as shown in Tables 4 and 12. Furthermore, the antibacterial effect of methanolic and ethanolic extracts of *P. ostreatus* and *P. florida* is more than that of aqueous extracts of the two species. In addition, it was also found that their effects with all extracts were found more on the methanolic extract than the others. A higher effect on the methanolic extract than on the others may be due to a higher diffusion rate of the extract into the methanolic than in the others, as shown in Tables 4, 8, and 12. The obtained results showed similarity with the findings of Menaga et al. [13]. In the present study, methanolic extracts of *Pleurotus* spp. showed the activity against *B. subtilis* (6.3 mm–15.5 mm). Coffee straw and *Sorghum* grain residue substrates provide the highest zone of inhibition, and aqueous extracts have showed the lowest zone of inhibition.

### 3.4. Effects on *Streptococcus faecalis*

The highest zone of inhibition was recorded in methanolic extracts of both *Pleurotus* species at the coffee straw and *Sorghum* grain residue (14.8 mm) in both species. The least zone of inhibition was recorded in aqueous extracts of pea straw (6.9 mm) of *P. Florida* species (Tables 5 and 13). This result was in agreement with the research conducted in [19].

### 3.5. Effects on *Pseudomonas aeruginosa*

All mushroom extracts have shown an antibacterial effect with the cultures of *Pseudomonas aeruginosa* on all substrates. Furthermore, the antibiotic effect of the coffee straw and *Sorghum* grain residue extract is more than that of the extracts of pea straw. In addition, it was also found that the zone of growth inhibition was found more on the coffee straw and *Sorghum* grain residue (Tables 6, 10, and 14).

Thus, selected mushroom species are a potentially good source of traditional medicines and therapeutics. The presence of bioactive compounds such as alkaloids, steroids, flavonoids, tannins, saponins, phenols, and phlobotannins in mushroom species can be used for the treatment of different ailments such as malaria, diarrhoea, skin burn, and antimicrobial agents. The antimicrobial activity showed the highest zone of growth inhibition by the coffee straw extract of *P. ostreatus* (16.4 mm) on *Pseudomonas aeruginosa*. It should be pointed out that the majority of the extracts present an antimicrobial activity against the tested bacterial species, which is in agreement with the results previously reported by our research group concerning *E. coli*, *K. pneumonia*, and *P. aeruginosa* [20], even when higher extract concentrations were used [21]. Nevertheless, a recent study describes methanolic extracts obtained from mushroom as having high antibacterial activity against *E. coli*, assessed by the disc diffusion method [22]. The extraction solvent or even the mushrooms' origin, as well as the bacterial strain, could explain the differences in the antimicrobial activity reported by different authors for the same species.

### 3.6. Effects on *Salmonella typhi*

Pea straw methanolic, ethanolic, and aqueous extracts of both mushroom species have showed the lowest antimicrobial effects on *Salmonella typhi *. The highest antimicrobial effect was recorded in methanolic extracts of *P. ostreatus* cultivated on the coffee straw substrate (16.9 mm, as shown on Table 7), while the lowest antimicrobial effect was noted in ethanolic extracts of pea straw (3.2 mm, as indicated in Table 11). The present study coincides with the work of Menaga et al. [13] which concluded that the methanol extract showed activity against *E. coli* (21 mm)*, Salmonella typhi* (20 mm)*, Staphylococcus aureus* (20 mm)*, Camphylobacter* spp. (19 mm)*, Bacillus* spp. (14 mm)*, Pseudomonas* spp. (8 mm)*, Klebsiella s*pp. (12 mm), and *Vibrio* spp. (20 mm). In the aqueous extract of *Pleurotus* spp.*, the highest effect on Salmonella typhi* was recorded by using coffee straw in both species (14.5 mm), and the lowest antibacterial effect was *observed in P. ostreatus* by using pea straw (4.6 mm) (Table 15).

### 3.7. Antibacterial Assay and MIC

The present study has revealed the antibacterial activity of the mushroom extract. The results of the antibacterial activity are presented in Table 16. All the mushrooms used in this study were found to exhibit various degrees of antimicrobial effects against the tested microorganisms. The zone of inhibition exhibited more than 10 millimeters was considered as highly active for extracts. *P. ostreatus* has a broad-spectrum antibacterial and antifungal activity. Similar antimicrobial potentials have been observed in the culture extracts of *Irpex lacteus* [23] and *Agrocybe* sp. [24, 25]. The best *in vitro* antibacterial activity was shown by *P. florida* (17 ± 0.2 mm) followed by *Pleurotus ostreatus*, and against *Esherichia coli*, *Pleurotus florida* also showed best antimicrobial activity than *Pleurotus ostreatus*. This antimicrobial efficacy of *Pleurotus florida* and *L. edodes* were similar to that in the study of Kuznetsov et al. [26]. Ishikawa et al. [27] reported data similar to the results obtained in this work, showing the antibacterial action of *Pleurotus florida* against *B. cereus*, *S. aureus*, and *E. coli*. Komemushi et al. [28] also reported that *L. edodes* and *P. florida* inhibited the growth of Gram-positive and Gram-negative bacteria. The revealed information regarding the strong antimicrobial activity of *P. ostreatus, P. florida*, *and Hypsizigus tessulatus* against pathogenic isolates has similarity to that in [29]. *Pseudomonas aeruginosa* showed more resistant to mushroom extracts comparatively than other isolates.

Among the bacterial isolates, the MIC of all mushroom extracts was high for *Pseudomonas aeruginosa*. The MICs of *P. florida* against all isolates were comparatively lower than those of *P .ostreatus*. The Lowest MIC was observed for *Salmonella typhi*, *Streptococcus faecalis*, *Esherichia coli*, and *P. florida*. This quite similar result was also observed by [21]. All the mushrooms also revealed an antimicrobial activity showing different MICs for each microorganism (Table 17).

## 4. Conclusions and Recommendation

### 4.1. Conclusions

The results of the study showed that the mushroom extracts indicate the presence of a potent antibacterial activity, which confirms its use against microbial pathogens including antibiotic-resistant bacteria. The results of these extracts for their antibacterial activities against 5 bacterial species showed high degrees of variation between the extraction solvents used and the species of test microorganisms. The extracts from the aqueous solvent had the lowest activity, indicating the possibility that the active antimicrobial components may not be of secretive nature. The results clearly indicated that the methanolic extracts from fruiting bodies had the widest spectrum and highest growth inhibitory effect against the test organism. The most sensitive bacterial species to this methanolic extract were *E. coli* (19.8 mm), *Salmonella typhi* (16.9 mm), *Pseudomonas aeruginosa* (16.4 mm), *B. subtilis* (15.5 mm), and *Streptococcus faecalis* (14.8 mm).

This result is in accordance with the fact that the methanolic extracts of fruiting bodies from both *Pleurotus ostreatus* and *Pleurotus florida* contained antibacterial compounds against certain fungal and bacterial pathogens [29].

The result obtained from the study is expected to show the antibacterial activity of *Pleurotus species* and which *Pleurotus species* has a high content of antibacterial activity so that they can be used to treat several bacterial diseases. So, the *Pleurotus species* are employed as an alternative source of medicine to mitigate the diseases associated with the microorganism.

In general, the resent finding encourages their use in human diets, which, in turn, might serve as protective agents to microbial diseases. Further researches are needed to be conducted in order to evaluate the antibacterial activity of other pathogenic bacterial species.

### 4.2. Recommendation

Out of selected mushroom species used for this study, all of them showed high antibacterial activity against the test pathogen, but there is limited scientific evidence on those mushroom species. So, further study is necessary to prove their potency. Further investigation is also needed to evaluate the effect of these mushrooms on a wide range of pathogenic bacteria.

## Figures and Tables

**Table 1:** Antibacterial activity of the methanolic extract of *Pleurotus* spp. cultivated on different substrates.

Bacteria used	Volume of extract per well (*μ*L)	Zone of inhibition (mm)									
		*P. ostreatus*				Control on NA	*P. florida*				Control on NA
		CS	SGR	PS	WG	Streptomycin	CS	SGR	PS	WG	Streptomycin
*E. coli*	20	14.5	14.3	7.3	8.4	26.2	12.2	11.3	9.6	7.7	25.6
	40	15.2	15.7	8.8	10.3	27.5	14.5	14.6	11.8	12.4	27.3
	60	16.8	16.1	10.5	11.5	28.9	16.4	16.7	12.6	14.5	28.5
	80	18.9	17.7	12.6	13.6	29.5	17.3	15.6	14.7	16.7	29.2
	100	19.5	19.8	16.4	17.5	29.8	17.9	16.9	17.4	18.6	29.7

Substrate: CS = coffee straw, SGR = *Sorghum* grain residue, PS = pea straw, WG = wheat grain, NA = nutrient agar.

**Table 2:** Antibacterial activity of the ethanolic extract of *Pleurotus* spp. cultivated on different substrates.

Bacteria used	Volume of extract per well (*μ*L)	Zone of inhibition (mm)									
		*P. ostreatus*				Control on NA	*P. florida*				Control on NA
		CS	SGR	PS	WG	Streptomycin	CS	SGR	PS	WG	Streptomycin
*E. coli*	20	15.9	12.3	8.3	8.6	25.4	10.2	10.3	8.5	8.4	24.8
	40	13.6	15.6	9.8	11.5	26.8	13.3	12.5	11.1	9.2	26.2
	60	15	16.3	11.5	10.4	27.6	14.7	14.2	11.8	11.7	28.5
	80	16.2	16.7	10.4	10.2	28.7	16.1	15.7	12.5	10.8	29.4
	100	17	17.6	12.5	10.7	29.4	18.4	17.9	14.4	12.5	29.8

Substrate: CS = coffee straw, SGR = *Sorghum* grain residue, PS = pea straw, WG = wheat grain, NA = nutrient agar.

**Table 3:** Antibacterial activity of the aqueous extract of *Pleurotus* spp. cultivated on different substrates.

Bacteria used	Volume of extract per well (*μ*L)	Zone of inhibition (mm)									
		*P. ostreatus*				Control on PDA	*P. florida*				Control on NA
		CS	SGR	PS	WG	Streptomycin	CS	SGR	PS	WG	Streptomycin
*E. coli*	20	12.9	9.4	8.3	7.6	24.3	7.2	9.1	9.3	8.7	23.7
	40	13.6	11.6	9.1	8.5	25.8	8.3	11.5	10.8	11.4	25.3
	60	14.2	12.3	10.5	10.5	27.8	10.6	12.1	12.4	12.5	27.5
	80	15.2	14.7	11.4	10.8	28.1	13.4	14.5	14.6	14.7	28.8
	100	16	15.6	12.0	11.7	29.6	16.4	17.1	17.4	16.6	29.9

Substrate: CS = coffee straw, SGR = *Sorghum* grain residue, PS = pea straw, WG = wheat grain, NA = nutrient agar.

**Table 4:** Antibacterial activity of the methanolic extract of *Pleurotus* spp. cultivated on different substrates.

Bacteria used	Volume of extract per well (*μ*L)	Zone of inhibition (mm)									
		*P. ostreatus*				Control on NA	*P. florida*				Control on NA
		CS	SGR	PS	WG	Streptomycin	CS	SGR	PS	WG	Streptomycin
*B. subtilis*	20	7.1	8.1	8.4	7.3	18	9.8	9.5	7.8	8.2	17
	40	8.8	10.5	9.3	8.4	19.8	10.4	10.6	9.2	9.4	22
	60	11.2	11	10.5	9.6	20.9	11.2	12.5	12.4	10.9	23.8
	80	12.7	11.8	11.3	11.6	22.6	12.4	13.1	12.9	12.7	24.9
	100	13.6	13.5	12.8	14.3	24.2	13.8	15.5	14.0	14.8	26.1

Substrate: CS = coffee straw, SGR = *Sorghum* grain residue, PS = pea straw, WG = wheat grain, NA = nutrient agar.

**Table 5:** Antibacterial activity of the methanolic extract of *Pleurotus* spp. cultivated on different substrates.

Bacteria used	Volume of extract per well (*μ*L)	Zone of inhibition (mm)									
		*P. ostreatus*				Control on NA	*P. florida*				Control on NA
		CS	SGR	PS	WG	Streptomycin	CS	SGR	PS	WG	Streptomycin
*Streptococcus faecalis*	20	9.1	8.6	7.5	7.7	16.4	8.3	7.3	6.9	8.1	15
	40	9.6	11.3	8.7	7.9	18.8	9.2	8.6	8.4	9.2	18.6
	60	10.8	11.9	9.6	8.4	21.4	10.5	9.7	9.5	9.9	20.2
	80	12.3	12.5	10.7	10.3	22.5	11.8	12.5	11.4	11.6	21.9
	100	14.8	12.9	13.9	13.5	23.6	14.6	14.8	13.7	14.3	22.4

Substrate: CS = coffee straw, SGR = *Sorghum* grain residue, PS = pea straw, WG = wheat grain, NA = nutrient agar.

**Table 6:** Antibacterial activity of the methanolic extract of *Pleurotus* spp. cultivated on different substrates.

Bacteria used	Volume of extract per well (*μ*L)	Zone of inhibition (mm)									
		*P. ostreatus*				Control on NA	*P. florida*				Control on NA
		CS	SGR	PS	WG	Streptomycin	CS	SGR	PS	WG	Streptomycin
*Pseudomonas aeruginosa*	20	10.2	9.3	9.7	8.7	18.2	9.2	9.4	10.2	10.2	17
	40	12.4	10.5	10.6	9.8	20.6	10.7	10.6	12.3	11.3	20
	60	14.5	12.6	12.4	12.5	23.5	13.4	12.7	13.4	13.4	22.7
	80	15.7	14.4	13.5	14.6	24.8	14.5	13.9	14.8	13.8	24
	100	16.4	15.6	14.2	15.3	26.9	16.2	14.5	15.5	14.8	26.3

Substrate: CS = coffee straw, SGR = *Sorghum* grain residue, PS = pea straw, WG = wheat grain, NA = nutrient agar.

**Table 7:** Antibacterial activity of the ethanolic extract of *Pleurotus* spp. cultivated on different substrates.

Bacteria used	Volume of extract per well (*μ*L)	Zone of inhibition (mm)									
		*P. ostreatus*				Control on NA	*P. florida*				Control on NA
		CS	SGR	PS	WG	Control on NA	CS	SGR	PS	WG	Control on NA
*B. subtilis*	20	7.5	7.1	7.4	7.3	17.2	7.8	8.5	8.2	7.9	16
	40	8.3	8.5	8.2	7.2	18.7	9.2	9.6	9.4	9.5	23
	60	10.0	10.5	10.6	8.7	20.3	10.2	11.6	11.4	11.5	24.4
	80	12.4	12.8	12.4	11.7	22.7	12.3	13.4	12.2	12.9	25.9
	100	12.8	13.2	13.4	15.3	23.9	13.7	14.9	14.8	13.0	26.8

Substrate: CS = coffee straw, SGR = *Sorghum* grain residue, PS = Pea straw, WG = wheat grain, NA = nutrient agar.

**Table 8:** Antibacterial activity of the ethanolic extract of *Pleurotus* spp. cultivated on different substrates.

Bacteria used	Volume of extract per well (*μ*L)	Zone of inhibition (mm)									
		*P. ostreatus*				Control on NA	*P. florida*				Control on NA
		CS	SGR	PS	WG	Streptomycin	CS	SGR	PS	WG	Streptomycin
*Streptococcus faecalis*	20	9.1	9.0	8.7	6.6	14.3	9.5	8.2	6.8	6.9	13
	40	8.5	8.8	8.4	7.9	16.2	10 .0	9.1	7.2	8.0	15.9
	60	10.1	11.2	9.2	9.4	16.9	11.4	10.1	8.8	9.2	16.1
	80	12.6	11.8	10.1	10.2	17.7	11.9	11.8	10.8	10.1	16.8
	100	13.9	13.2	10.9	12.3	19.9	12.3	12.1	11.9	13.2	19.2

Substrate: CS = coffee straw, SGR = *Sorghum* grain residue, PS = pea straw, WG = wheat grain, NA = nutrient agar.

**Table 9:** Antibacterial activity of the ethanolic extract of *Pleurotus* spp. cultivated on different substrates.

Bacteria used	Volume of extract per well (*μ*L)	Zone of inhibition (mm)									
		*P. ostreatus*				Control on NA	*P. florida*				Control on NA
		CS	SGR	PS	WG	Streptomycin	CS	SGR	PS	WG	Streptomycin
*Pseudomonas aeruginosa*	20	9.4	9.1	8.8	9.3	15.8	8.7	8.4	9.2	9.0	15
	40	10.3	9.8	9.4	9.9	17 .5	9.7	9.6	11.4	10.3	17
	60	12.8	11.4	11.6	12.5	19.9	12.8	12.2	13.2	12.7	19.3
	80	13.9	12.6	13.1	12.7	22	13.6	13.7	14.1	13.2	21
	100	14.8	15.2	14.4	15.6	24.3	14.8	14.4	15.0	14.1	23.4

Substrate: CS = coffee straw, SGR = *Sorghum* grain residue, PS = pea straw, WG = wheat grain, NA = nutrient agar.

**Table 10:** Antibacterial activity of the ethanolic extract of *Pleurotus* spp. cultivated on different substrates.

Bacteria used	Volume of extract per well (*μ*L)	Zone of inhibition (mm)									
		*P. ostreatus*				Control on NA	*P. florida*				Control on NA
		CS	SGR	PS	WG	Streptomycin	CS	SGR	PS	WG	Streptomycin
*Salmonella typhi*	20	9.4	9.8	3.2	8. 8	17.1	9.2	9.5	3.7	8. 6	16
	40	10.6	10.7	5.1	9.5	17.9	10.9	10.3	9.3	9.4	17.2
	60	12.5	13.5	10.2	10.5	18.8	11.6	11.2	9.9	10.6	18.1
	80	12.8	14.1	11.3	12.4	19.5	12.7	12.4	10.5	12.3	19.3
	100	15.4	14.5	11.8	14.3	22.6	14.3	13.8	12.1	14.6	21.7

Substrate: CS = coffee straw, SGR = *Sorghum* grain residue, PS = pea straw, WG = wheat grain, NA = nutrient agar.

**Table 11:** Antibacterial activity of the aqueous extract of *Pleurotus* spp. cultivated on different substrates.

Bacteria used	Volume of extract per well (*μ*L)	Zone of inhibition (mm)									
		*P. ostreatus*				Control on NA	*P. florida*				Control on NA
		CS	SGR	PS	WG	Streptomycin	CS	SGR	PS	WG	Streptomycin
*B. subtilis*	20	6.7	7.0	6.6	6.3	18.3	7.7	7.5	7.2	6.9	17
	40	8.1	8.6	7.2	8.2	19.7	9.1	8.5	8.4	8.5	21.7
	60	9.0	9.5	9.6	9.5	20.2	9.4	11.4	10.3	11.5	23.4
	80	11.5	11.3	12.3	10.7	22.3	11.5	13.2	12.5	12.7	25.0
	100	12.6	13.5	13.7	12.3	25.8	13.8	14.7	13.8	13.6	27.2

Substrate: CS = coffee straw, SGR = *Sorghum* grain residue, PS = pea straw, WG = wheat grain, NA = nutrient agar.

**Table 12:** Antibacterial activity of the aqueous extract of *Pleurotus* spp. cultivated on different substrates.

Bacteria used	Volume of extract per well (*μ*L)	Zone of inhibition (mm)									
		*P. ostreatus*				Control on NA	*P. florida*				Control on NA
		CS	SGR	PS	WG	Streptomycin	CS	SGR	PS	WG	Streptomycin
*Streptococcus faecalis*	20	6.2	7.0	7.2	6.6	13.1	8.3	8.1	6.9	6.4	12
	40	7.6	8.6	8.8	7.9	14.7	10.0	8.7	7.5	8.0	14.0
	60	9.4	11.4	9.0	8.2	15.8	11.2	9.1	8.4	9.1	16.1
	80	12.7	12.6	10.0	10.7	16.7	11.7	11.7	11.0	10.1	17.6
	100	14.5	13.9	11.5	12.4	19.2	12.2	12.6	11.1	13.2	18.4

Substrate: CS = coffee straw, SGR = *Sorghum* grain residue, PS = pea straw; WG = wheat grain, NA = nutrient agar.

**Table 13:** Antibacterial activity of the aqueous extract of *Pleurotus* spp. cultivated on different substrates.

Bacteria used	Volume of extract per well (*μ*L)	Zone of inhibition (mm)									
		*P. ostreatus*				Control on NA	*P. florida*				Control on NA
		CS	SGR	PS	WG	Streptomycin	CS	SGR	PS	WG	Streptomycin
*Pseudomonas aeruginosa*	20	8.2	7.1	7.3	7.2	13.1	7.7	8.5	7.9	9.2	12
	40	9.1	8.8	8.1	8.9	14.5	8.7	9.4	8.6	9.3	15.4
	60	10.6	9.2	10.4	11.5	15.5	10.5	11.5	12.7	10.5	17.6
	80	12.4	12.2	12.2	13.7	21.3	12.4	13.4	14.5	12.4	20.1
	100	13.2	14.6	14.5	14.3	22.9	13.9	14.2	15.2	14.5	22.7

Substrate: CS = coffee straw, SGR = *Sorghum* grain residue, PS = pea straw; WG = wheat grain, NA = nutrient agar.

**Table 14:** Antibacterial activity of the aqueous extract of *Pleurotus* spp. cultivated on different substrates.

Bacteria used	Volume of extract per well (*μ*L)	Zone of inhibition (mm)									
		*P. ostreatus*				Control on NA	*P. florida*				Control on NA
		CS	SGR	PS	WG	Streptomycin	CS	SGR	PS	WG	Streptomycin
*Salmonella typhi*	20	8.2	7.8	4.6	7. 6	14.1	8.4	8.5	6.2	8. 7	13.2
	40	9.5	8.6	5.3	8.4	14.9	9.7	9.4	9.2	9.2	14.3
	60	11.4	11.6	9.2	9.3	15.7	10.4	10.3	9.4	10.1	15.9
	80	12.7	12.4	10.6	10.2	16.7	12.4	11.5	10.2	11.2	16.8
	100	14.5	13.5	11.5	12.3	18.2	14.5	13.2	11.5	13.4	17.9

Substrate: CS = coffee straw, SGR = *Sorghum* grain residue, PS = pea straw, WG = wheat grain, NA = nutrient agar.

**Table 15:** Antibacterial activity of the methanolic extract of *Pleurotus* spp. cultivated on different substrates.

Bacteria used	Volume of extract per well (*μ*L)	Zone of inhibition (mm)									
		*P. ostreatus*				Control on NA	*P. florida*				Control on NA
		CS	SGR	PS	WG	Streptomycin	CS	SGR	PS	WG	Streptomycin
*Salmonella typhi*	20	10.3	10.4	5.3	9.9	17.8	10.3	10.5	4.7	9. 6	17
	40	11.4	11.5	9.5	10.5	19.2	10.9	11.7	5.3	9.8	19
	60	12.6	13.0	10.4	11.4	22.7	12.4	12.2	10.2	10.7	22.3
	80	12.9	13.3	11.3	14.3	23.6	13.2	13.1	11.8	12.1	23.7
	100	16.9	14.5	12.5	15.2	25.8	15.5	13.9	12.9	14.8	25.4

Substrate: CS = coffee straw, SGR = *Sorghum* grain residue, PS = pea straw, WG = wheat grain, NA = nutrient agar.

**Table 16:** Preliminary antibacterial testing of mushroom methanolic extracts through determination of the zone of inhibition (mm ±SD)*∗*.

Isolates	Mushroom extracts		Standard drug
	*P. ostreatus*	*P. florida*	Streptomycin
*Esherichia coli*	14 ± 0.2	17 ± 0.2	24 ± 0.2
*Bascillus subtilis*	13.5 ± 0.1	16 ± 0.2	25 ± 0.2
*Streptococcus faecalis*	13.4 ± 0.1	14 ± 0.1	25 ± 0.2
*Pseudomonas aeruginosa*	7 ± 0.2	12 ± 0.2	20 ± 0.1
*Salmonella typhi*	11.5 ± 0.3	15 ± 0.1	20 ± 0.1

^*∗*^The diameters of the zone of inhibition are expressed in millimeter (mm) as mean ± standard deviation (SD).

**Table 17:** Minimum inhibitory concentrations determination of extracts.

Isolates	Mushroom extracts	
	*P. ostreatus*	*P. florida*
*Esherichia coli*	7	6
*Bascillus subtilis*	8	7
*Streptococcus faecalis*	7	6
*Pseudomonas aeruginosa*	9	8
*Salmonella typhi*	7	5

*Note*. Each value is expressed as mean (*n* = 3).

## Data Availability

The dataset used to support the findings of this study is available from the corresponding author upon request.
